# A Model of Evolutionary Selection: The Cardiovascular Protective Function of the Longevity Associated Variant of BPIFB4

**DOI:** 10.3390/ijms19103229

**Published:** 2018-10-19

**Authors:** Francesco Villa, Albino Carrizzo, Anna Ferrario, Anna Maciag, Monica Cattaneo, Chiara Carmela Spinelli, Francesco Montella, Antonio Damato, Elena Ciaglia, Annibale Alessandro Puca

**Affiliations:** 1 Cardiovascular Research Unit, IRCCS MultiMedica, 20138 Milan, Italy; francesco.villa@multimedica.it (F.V.); anna.ferrario@multimedica.it (A.F.); anna.maciag@multimedica.it (A.M.); monica.cattaneo@multimedica.it (M.C.); spinellichiara82@gmail.com (C.C.S.); 2 IRCCS Neuromed, Pozzilli, 86077 Isernia, Italy; albino.carrizzo@gmail.com (A.C.); antonio.damato85@libero.it (A.D.); 3 Department of Medicine, Surgery and Dentistry “Scuola Medica Salernitana”, University of Salerno, Via Salvatore Allende, 84081 Baronissi, Italy; f.montella89@gmail.com (F.M.); eciaglia@unisa.it (E.C.)

**Keywords:** BPIFB4, aging, cardiovascular disease, angiogenesis, eNOS

## Abstract

Evolutionary forces select genetic variants that allow adaptation to environmental stresses. The genomes of centenarian populations could recapitulate the evolutionary adaptation model and reveal the secrets of disease resistance shown by these individuals. Indeed, longevity phenotype is supposed to have a genetic background able to survive or escape to age-related diseases. Among these, cardiovascular diseases (CVDs) are the most lethal and their major risk factor is aging and the associated frailty status. One example of genetic evolution revealed by the study of centenarians genome is the four missense Single Nucleotide Polymorphisms (SNPs) haplotype in bactericidal/permeability-increasing fold-containing family B, member 4 (BPIFB4) locus that is enriched in long living individuals: the longevity associated variant (LAV). Indeed, LAV-BPIFB4 is able to improve endothelial function and revascularization through the increase of endothelial nitric oxide synthase (eNOS) dependent nitric oxide production. This review recapitulates the beneficial effects of LAV-BPIFB4 and its therapeutic potential for the treatment of CVDs.

## 1. Introduction

The World Health Organization recognizes Cardiovascular Diseases (CVDs) as the deadliest condition in the world, with 17.7 million people dead in 2015, 31% of all global deaths [1]. CVDs are associated with several risk factors: behavioral (tobacco and alcohol consumption, physical inactivity, unhealthy diet), environmental and pathological (diabetes, immunological disorders) [2,3,4,5,6,7,8].

In the last century, the sedentary lifestyle together with wrong nutritional habits and air pollution led to an increased incidence of CVD-related morbidity and mortality.

A population study performed in countries suffering from financial crisis revealed a strong association between low socioeconomic status and CVDs, demonstrating that the progress in medicine sciences does not impact CVDs’ incidence equally for all people [9].

However, the risk factor with the biggest impact is the progressive aging of population. Aging exposes individuals to age-related disorders like hypertension, diabetes, elevated cholesterol levels and to lifelong harmful effects, like prolonged use of tobacco, obesity and physical inactivity [10]. The recent improvement in healthcare and medical innovations have reduced mortality in young and middle aged people and increased life-expectancy [11]. People live longer and longer throughout the world, but the increase in lifespan does not coincide with increase in health-span, i.e., the duration of disease-free life. The result is that society has to face an increasing number of people with disabilities, for the most part cardiovascular and cerebrovascular diseases, as indicated in the United Nations’ report [12].

Aging cannot be avoided but at most delayed. The goal is to minimize aging-related negative and cumulative effects and limit CVD disabilities, allowing people to live a healthy aging [13]. This is the case of long-living individuals, which delay or avoid diseases of aging living more than 20 years longer than what was expected for the general population.

## 2. Genome-Wide Association Studies: Healthspan Depends on a Vascular System Condition

The centenarian population around the world represents a natural genomic model that has started to be extensively analyzed in order to understand the molecular mechanisms for a good aging. It is known that longevity is the resultant of the interaction between multiple factors, among which the genetic component has a huge impact. The mortality rate of centenarians’ siblings is halved throughout the adult life [14]. Furthermore, offspring of centenarians show a lower incidence of cancer- and cardiovascular-related mortality (71% and 82% lower risk, respectively) [15,16].

In particular, Genome-Wide Association Studies (GWAS) discovered polymorphic gene variants linked to the longevity trait, possibly thanks to their beneficial effects on aging. Many polymorphisms associated with longevity are unique for a specific human population; others are found in different populations in different countries. 

As a relevant example, Apolipoprotein E (APOE) isoform epsilon 4 is negatively linked to longevity for being responsible for mortality of stem cell pool, and by causing Alzheimer’s Disease (AD) and worsening CVD outcome. Recently, a meta-analysis revealed a protective role for the APOE isoform epsilon 2 [17].

Furthermore, a family based study has identified elongation of very long chain fatty acids protein 6 (ELOVL6) to reside in q25, a locus linked to exceptional longevity [18,19]. The association of APOE and ELOVL6 with longevity is probably due to through their role in cardiovascular system and metabolism. Indeed, APOE epsilon 2 is associated with a reduced incidence of atherosclerosis [17,20,21], while ELOVL6 is an enzyme that catalyzes the elongation of the fatty acids chain and its downregulation leads to increased insulin sensitivity [22]. Many studies point to a role of fatty acids in establishing cardiovascular outcome. As an example, high poly-unsaturated fatty acid (PUFA) and low mono-unsaturated fatty acid (MUFA) of erythrocytes associate with Atrial Fibrillation or Atrial Flutter [23].

Lipid composition of cell membranes, impacting oxidative intracellular stress, influences lifespan in humans [24,25]. 

An additional gene belonging to the 4q25 locus, S-nitrosoglutathione reductase (GSNOR), regulates *S*-nitrosylation, a redox-based posttranslational modification dysregulatyed in pathological conditions [26]. GSNOR expression is decreased during aging while it is preserved in long living individuals’ (LLIs) mononuclear cells (MNCs). It is involved in the correction of age-related defects in mitochondrial physiology caused by epigenetic deregulation. Another association with longevity has been found with a Single Nucleotide Polymorphism (SNP) located in Forkhead box O3A (FOXO3A). This gene plays a fundamental role in preventing oxidative stress damages and regulating autophagic flux, which, when impaired, may cause several neoplastic conditions [27,28]. 

## 3. Longevity Associated Variant (LAV) of Bactericidal/Permeability-Increasing Fold-Containing Family B, Member 4 (BPIFB4)

We have recently performed a GWAS on LLIs and controls in a centenarian population living in Southern Italy (Southern Italian Centenarian Study) through a case-control study design. The results highlighted 67 SNPs potentially involved in determining exceptional longevity that showed a *p* < 0.0001 under different genetic models [29]. Among the top findings, four SNPs satisfied the statistical filtering, i.e., they were missense changes with a better chance to be a true positive because of the potential biological impact. Of these four, only one was significantly replicated under a recessive genetic model in other two independent populations from Germany (German Longevity Study, [30]) and the USA [31]. In conclusion, homozygous for the minor allele of rs2070325 SNP were significantly enriched in LLIs and in linkage disequilibrium with the other three SNPs (rs2889732, rs11699009, and rs11696307), all non-synonymous aminoacidic changes: Ile229Val, Asn281Thr, Leu488Phe, and Ile494Thr. The resultant 4-SNPs haplotype lies in the functionally unknown BPIFB4 gene, and it arranges a particular variant of the protein, the longevity associated variant (LAV-BPIFB4), which was revealed to have important beneficial effects in many molecular pathways (see below). LAV-BPIFB4 has an allele frequency of 29.5%, while the wild type isoform (WT-BPIFB4) allele frequency is around 66% in the Caucasian population. Homozygous genotype reaches 14% in centenarians as compared to 10% in young controls.

## 4. Aging and Cardiovascular Diseases

Aging affects vessels under many aspects. Aged vessels are structurally compromised and present augmented stiffness and frailty. Moreover, there is an important impairment of stem cells availability and an almost total exhaustion of endothelial progenitor cell niches, leading to a deranged vascular regeneration and repair. Furthermore, aged vessels contain a low level of Nitric Oxide (NO) secondary to a diminished activity of Nitric Oxide Synthase (NOS). NO is a very important molecule for endothelial activity and it acts as cofactor in many molecular processes linked to angiogenesis and vascularization [32].

Aging causes a deep transformation of the entire cardiovascular system, including heart [33]. Thus, it is crucial to understand the molecular mechanisms that compromise the cardiovascular system during aging and how to fix them. Managing the aging process of cardiovascular system may increase lifespan and improve life quality in elderly people. In particular, we could prevent the onset of acute episodes, such as stroke or myocardial infarction, but also have a better outcome from those events and retard and mitigate chronic disorders.

A generally accepted hypothesis is that the immune system is critically involved in the pathogenesis of CVDs. Leukocytosis and monocytosis have been associated with CVDs in numerous epidemiological studies, prompting to speculate on the functional importance of these cells [34]. Indeed, mononuclear phagocytic cells (MPS) are essential players in the fine orchestration of tissue repair and functional recovery within the cardiovascular system. Their function is to “patrol” endothelial maintenance in the circulation and tissue homeostasis by differentiating into a broad spectrum of either inflammatory (M1) or resolving (M2) macrophages [35]. Elderly people are subjected to an improper balance between pro- and anti-inflammatory immune responses. Usually, aging is characterized by a prevalence of inflammation, a phenomenon called inflammaging [36,37] that can compromise several districts in the body, especially the cardiovascular system. As a proof of concept, CVDs have a higher incidence in people affected by chronic inflammatory diseases (CID). This association is due to the key role of monocytes and other immune system players in determining inflammation condition and promoting pathological events such as atherosclerotic plaques formation and loss of cellular homeostasis [38,39].

Vessels are complex structures able to control blood flow and blood pressure through different stimuli, which are finely tuned. During aging, the fine-tuning is lost with a general reduction in vasodilatatory capacity of vessels resulting in cardiovascular hypertension. This condition is due to a decrease in vasorelaxant stimuli, a substitution of an elastin component of the media arterial wall with collagen and calcium deposit [33].

Vasorelaxation is mostly due to the activity of eNOS, an enzyme constitutively expressed in endothelial cells (EC) and circulating MNCs that catalyzes NO in both a calcium dependent and independent manner, producing l-arginine with the consumption of NADPH. eNOS activity is regulated by phosphorylation, dimerization, and interaction with co-factors, such as tetrahydrobiopterin (BH4) [40,41].

**Figure 1:**
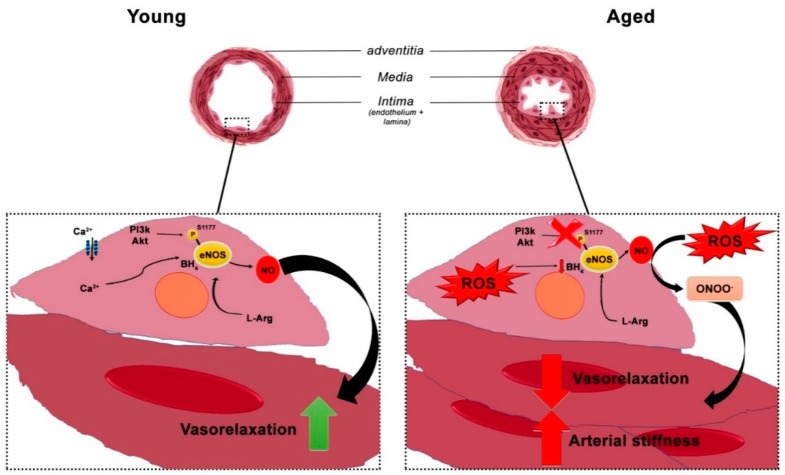
Age-induced arterial dysfunction. (**left**): young elastic artery with a thin structure and functional nitric oxide synthase that result in a proper vasorelaxation (green arrow); (**right**): older artery with thickened intima and media and dysfunctional nitric oxide synthase that promote cardiovascular pathological conditions, such as the decrease of vasorelaxation ability and the increase of stiffness (red arrows).

NO lack of bioavailability can be caused by many pathological conditions, such as diabetes, obesity, hypertension, hyperlipidemia, atherosclerosis, all age-related traits. The progressive decrement of NO during aging is primarily due to the increase of oxidative stress that causes the uncoupling of eNOS and a decrease of its cofactor BH4 expression. This modulation brings to the formation of peroxynitrite (ONOO^−^), a strong oxidant that promotes the progressive arterial stiffness and microvascular dysfunction (Figure 1). Recent studies have revealed the contribution of endoplasmic reticulum stress in the imbalance between reactive oxygen species (ROS) production and antioxidant responses, which causes endothelial dysfunction. The unfolded protein response (UPR) pathways play an essential role. Indeed, in the attempt to improve proteostasis, the persistent activation of UPR, as occurs during aging, may lead to increased oxidative stress and cell death. This molecular switch has been also correlated to the onset or to the exacerbation of the endothelial dysfunction in cardiovascular diseases, making UPR a potential target for the clinical management of this aging condition [42].

Furthermore, during aging, vessels become progressively unable to maintain blood flow, due to loss of angiogenic ability [43].

As mentioned before, calcium ions are crucial for eNOS activation. Furthermore, calcium homeostasis is important for the achievement of healthy aging because of its role in many signal transduction pathways, neurotransmission processes, muscle contraction, T cell activation and cell structure stability [44,45].

## 5. The Role of Endothelial Cells in Vascular Homeostasis

ECs are usually quiescent under physiological conditions while entering a proliferation state under pathological stimuli that induce wound healing, vascular inflammation, and tumourigenesis [46]. Senescence is an irreversible process that impairs EC capability to release NO. Thus, the correction of EC senescence process may represent a therapeutic target for improving beneficial activity of ECs in vessels.

Indeed, the senescence process can be slowed by proteins (such as kallistatin [47]) or non-coding RNAs (such as microRNAs [48]). Another way to obtain the same protective effect is the stimulation of resident ECs, which can be operated by endothelial progenitor cells (EPCs) [49] the most significant reservoir of endothelial circulating stem cells involved in vascular homeostasis in physiological conditions. Specifically, EPCs sustain the adult neovascularization during cardiovascular injury, by either engraftment-based mechanisms into the damaged vessels or more importantly by secreting a wide range of vasoactive substances including matrix proteins, growth factors and cytokines. Aging is known to influence the EPC function and number, by negatively impacting the physiological endothelial performance of older adults. Recent studies demonstrated that endothelial cells participate in both innate and adaptive immune responses by detecting blood circulating endogenous signals, acting as a conditional antigen presenting cells and immune cell mobilizers and by functioning as immune/inflammation effectors [50]. As such, we can speculate that EPC protective functions might also be negatively affected by the sustained low grade of inflammation typical of the elderly.

## 6. LAV-BPIFB4 Characterization and Its Therapeutic Potential

### 6.1. Structure and Localization

**Figure 2:**
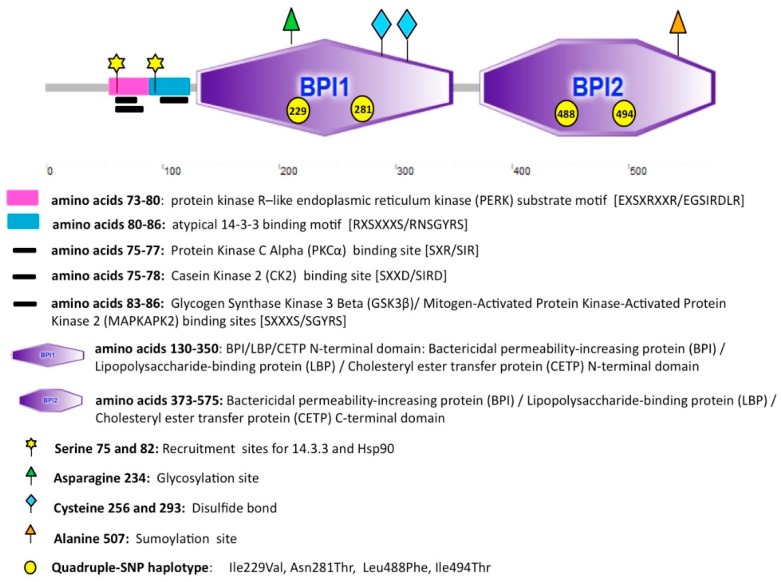
Schematic representation of domains organization and post-translational modifications in bactericidal/permeability-increasing fold-containing family B, member 4 (BPIFB4). The figure shows BPIFB4 structure and indicates the position of phosphorylation sites, the binding sites and the post translational modifications [52].

BPIFB4, in full: bactericidal/permeability-increasing fold-containing family B, member 4, protein is expressed in olfactory epithelium, MNCs, germline, stem, progenitor, and fetal cells. The structure of BPIFB4 includes a regulatory domain (N-terminal) containing phosphorylation and binding sites and two large binding pockets (indicated in Figure 2 as BPI1 and BPI2) [51].

While the WT-BPIFB4 isoform shows a nuclear localization, the LAV-BPIFB4 isoform is more prone to protein kinase R (PKR)-like endoplasmic reticulum kinase (PERK) dependent phosphorylation and is assembled in a complex with 14.3.3 and Heat Shock Protein 90 (HSP90), which is retained in the cytoplasm. The PERK kinase pathway is involved in an unfolded protein response and the involvement of BPIFB4 indicates its possible role in reducing endoplasmic reticulum stress and improving cells’ homeostasis [29].

### 6.2. Molecular Functions and Cardiovascular Therapeutic Role

Individuals carrying the protective variant of BPIFB4 in homozygosis are shown to have much higher levels of phosphorylated eNOS in MNC compared to the non-carriers (heterozygous or wild-type subjects). As mentioned before, higher eNOS activation brings many beneficial effects on the physiological functions of the vascular system. Indeed, the perfusion of plasmids carrying LAV-BPIFB4 cDNA in ex-vivo mouse arteries enhances their ability to react to vasorelaxant stimulus acetylcholine and increases eNOS activation through Serine 1177 phosphorylation. On the contrary, siRNA mediated knockdown of BPIFB4 causes eNOS inactivation and a striking impairment in vessels’ activity. In the presence of eNOS inhibitor *N*^G^-nitro-l-arginine methyl ester (L-NAME) and PERK inhibitor GSK2606414, LAV-BPIFB4 loses its protective effect. Mutagenesis removing LAV-BPIFB4 binding site for 14.3.3 and the ser 75 phosphorylation site for PERK also blocked the protein action [29].

BPIFB4 beneficial effect is even more appreciable when the overexpression of the protective variant is performed in vivo, by injecting animal models with adenoviral vectors. LAV-BPIFB4 enhances vessel activity and decreases blood pressure both in wild type mice strains and in hypertensive rats, highlighting its therapeutic potential. Notably, LAV-BPIFB4 is able to rescue eNOS phosphorylation and vessel activity in 22–24 months old dysfunctional mice to levels observable in young mice [29].

### 6.3. Endothelial Progenitor Cells Homing Enhancement

LAV-BPIFB4 has a very important role in supporting the homing of stem cells or endothelial precursor cells and recovering the repairing and regeneration abilities of aged and ischemic vessels. As mentioned before, during aging, the bioavailability of endothelial progenitor cells, indispensable for the maintenance of vessel integrity through regeneration, is reduced [29].

LSK cells (Lin^−^ Sca-1^+^ cKit^+^) are a subset of MNC isolated from peripheral blood that have a marked vessels regeneration and repair ability. LAV-BPIFB4 promotes LSK cell homing and ability to rescue blood flow in mouse compromised ischemic limbs, through a revascularization process that significantly increases both capillary and arteriole density. The capability of LAV-BPIFB4 to enhance pro-angiogenesis makes this protein a candidate as a powerful therapeutic tool for limb-ischemia in different conditions, including diabetes.

Furthermore, the BPIFB4 RNA level is higher in centenarians’ CD34^+^ cells as compared to young controls. CD34^+^ cells represent a MNC subset enriched of proangiogenic progenitors thus indicating a possible protein role in modulating vessels repair process. Silencing of BPIFB4 in EPCs produces a loss of their migration ability upon SDF1 stimulation, while LAV-BPIFB4 overexpression significantly increases homing of progenitor cells, enhancing blood flow, and spontaneous revascularization, fundamental processes for delaying the aging of the endothelium [29]. 

### 6.4. Enhancement of Calcium Mobilization

A more recent study, aimed at understanding the molecular players involved in BPIFB4 protective action, showed that the LAV isoform acts by increasing calcium mobilization through the phosphorylation of protein kinase C alpha (PKCα) [53]. LAV-BPIFB4, indeed, enhances PKCα translocation to the membrane, a crucial step for its activation. Furthermore, BPIFB4 contains a phosphorylation site (Ser 75) for PKCα that in turn is activated by LAV-BPIFB4 through calcium mobilization [53]. Consistently, LAV-BPIFB4 loses its protective role in the absence of extracellular calcium. Taken together, these results collocate BPIFB4 between PKCα and eNOS and define PKCα and Calcium as fundamental cofactors for exhibiting its potential. 

Moreover, the beneficial effect of LAV-BPIFB4 can be wider than expected because the mobilization of calcium is involved in the etiopathogenesis of a diversified degree of age-related diseases, such as cardiac disorders, neuro-muscular syndromes and neurological diseases.

### 6.5. Alternative BPIFB4 Isoforms

LAV-BPIFB4 is not the only variant that has demonstrated beneficial effects for healthy aging. The expression of BPIFB4 WT isoform is found increased in serum and in MNCs of healthy aged individuals [54,55]. In the presence of low nutrient/low oxygen conditions, MNCs of centenarian people with no history of disease express a higher level of BPIFB4 and show higher activity of C-X-C chemokine receptor type 4 (CXCR4), the chemokine receptor responsible for MNC migration.

Moreover, we also discovered the presence of another BPIFB4 variant in 4% of chromosomes. This protein is the result of a recombination event that generates a chimera of the WT and LAV isoform and that only retains the mutations in position 3 and 4 of the 4 SNPs haplotype [56]. The overexpression of this BPIFB4 rare variant (RV) in mouse models impairs phosphorylation of eNOS and blunts acetylcholine-mediated vasorelaxation. Furthermore, RV-BPIFB4 is able to induce hypertension when expressed in mice through adenoviral vector injection. Accordingly, a population-based study found that RV-BPIFB4 carriers have a significantly higher blood pressure as compared to the other individuals among the people under anti-hypertensive treatment, suggesting that the rare variant may be used as a novel biomarker of vascular dysfunction. On the other side, individuals’ homozygote for the minor allele rs2070325 have a significantly lower diastolic blood pressure (DBP) when compared to major allele carriers, and a slight decrease of systolic blood pressure (SBP) [56]. These clinical findings reflect the ex-vivo experiments, where the overexpression of the LAV-BPIFB4 isoform improved vascular function, while the siRNA mediated silencing of BPIFB4 or the overexpression of its RV isoform produced detrimental effects on vascular activity [29,56].

### 6.6. Molecular Mechanism of BPIFB4 Isoforms

**Figure 3:**
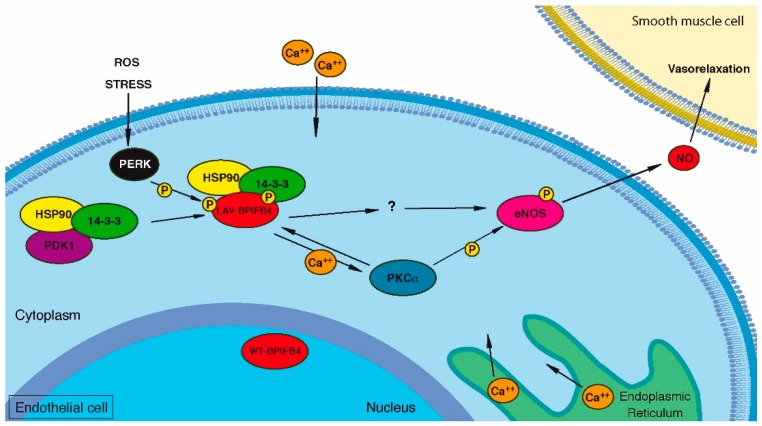
Schematic mechanism of function of Longevity Associated Variant (LAV) of BPIFB4. The figure shows the schematic representation of the pathway that involves BPIFB4. The LAV isoform is retained in the cytoplasm by the enhanced interaction with 14-3-3 protein and it is phosphorylated by protein kinase R (PKR)-like endoplasmic reticulum kinase (PERK) and protein kinase C alpha (PKCα). This activation leads to the phosphorylation of endothelial nitric oxide synthase (eNOS) through PKCα and through still unknown players. eNOS produces NO that is used for the activity of smooth muscle cells. Small yellow “P” circles indicate phosphorylation events.

The observations about the three main isoforms of BPIFB4 (WT, LAV and RV) suggest the following mechanism: BPIFB4 assembles in a complex with heat shock protein 90 (HSP90) and 14.3.3 proteins, and it is activated by PERK stress kinase-dependent phosphorylation at Serine 75. Under this conformation, BPIFB4 triggers eNOS phosphorylation at Serine 1177 and the production of NO in the endothelium. This mechanism is mediated by calcium mobilization that causes the activation of PKCα and, in turn, PKCα-mediated the activation of BPIFB4 [53] (Figure 3). One of the differences between the three isoforms is their subcellular localization: WT-BPIFB4 is mainly nuclear, LAV isoform is mainly cytoplasmic, while RV isoform can be both [29]. The prevalent cytoplasmic localization of LAV-BPIFB4, due to the structural conformation imposed by mutations, allows a better interaction of BPIFB4 with its partners HSP90 and 14.3.3. Moreover, this conformation enhances calcium entry in the cytoplasm, the consequent activation of PKCα and its effect on BPIFB4. The other isoforms have a poor (WT) or detrimental (RV) impact in this process.

## 7. Therapeutic Approaches

CVDs produce a variety of pathological consequences to the heart, (Coronary Heart Disease, Myocardial Infarction and Heart Failure), the brain (Cerebral Ischemia), and to the limb (Limb Ischemia). All of these outcomes occurring after an arterial occlusion compromising the oxygen supply to the organ, are critical and often irreversible conditions leading to the failure of the damaged organ. For this reason, medicine research aims at developing a treatment able to preserve blood flow and contrast the first phases of ischemic occlusion through spontaneous angiogenic processes.

Angiogenesis is driven by ECs that are activated in the presence of proangiogenic factors and move toward the ischemic zone [57]. Furthermore, physical and chemical stimuli drive the bone marrow to release EPCs CD34^+^ with a major role in the activation of resident ECs [49].

Nowadays, two major therapeutic approaches are in clinics: proangiogenic factor administration and cell therapy. In the first case, administration of Vascular Endothelial Growth Factor-A (VEGF-A) or Fibroblast Growth Factor 2 (FGF2) showed some good results, but only in preclinical studies. These factors are able to induce angiogenesis and improve vascular activity through activation of eNOS, but their administration during clinical trials did not show any efficacy, also in the case of gene therapy-mediated administration [58].

Another therapeutic approach has the aim to recover functional eNOS coupling. Treatment with antioxidant combined to BH4 were experimented [59,60,61], but the best results were obtained with statins, supported by cofactors administration or other NO donor drugs ad hoc developed. These molecules are able to increase transient NO generation through the activation of protein kinase B (AKT) and protein kinase A (PKA) [62] and to enhance the activation of circulating proangiogenic cells [63,64,65].

Overall, since most of the therapeutic approaches are based on improving NO release and angiogenesis, which are also induced by LAV-BPIFB4, we candidate this protein as a novel therapeutic tool.

## 8. Microvasculopathies

In the attempt to envision new therapeutic strategies, it is important to discriminate between macro- and micro-vasculopathies. Even though vasculopathies have common causes, statins are usually powerless against micro-vasculopathies, which affects small vessels like capillaries. One of the most affected organs is a very delicate and sensible part of the eye, the retina, which can be compromised by ischemic events.

Premature birth represents one of the most common causes of retinal vasculopathy [66,67,68]. The retinopathy of prematurity (ROP) is not caused by low oxygen levels, but, instead, it is due to the exposure to supraphysiological levels of oxygen, provided to a premature newborn. In hyperoxic conditions, VEGF, insulin-like growth factor 1 (IGF-1) and stromal cell-derived factor 1 (SDF1), usually induced by hypoxia, are all downregulated [69,70,71]. The loss of these vasogenic factors causes a delay in physiological development of retinal vessels and predisposes the eye to the first phase of the ROP. The second phase is characterized by the return of the tissues to physiological oxygen levels. The retina feels this change like an actual hypoxic environment and goes through an abnormal process of neovascularization. VEGF and others hypoxia-induced factors (HIFs) are upregulated. VEGF, in particular, is responsible for the blood–retina barrier dysfunction, a typical disorder linked to serious ROP [72,73]. For this reason, in order to find a treatment, it is important to find a way to control HIFs and other factors that may influence down-stream cellular signals, such as oxygen level, inflammation and nutritional status [67]. A possible mechanism for modulating HIF and VEGF pathways is DNA methylation that affects expression of genes involved in antioxidant response, hypoxic stress and inflammation. These mechanisms have been associated to age-related macular degeneration [74,75].

There are two main strategies for the treatment of ROP: surgical ablation and pharmaceutical/cell-based therapy. Cryosurgery ablates the area of the retina that is not vascularised in order to avoid the abnormal vascular neo-formation led by hypoxia. This strategy is not very effective as it reduces the risk of blindness by only 25% [68,76,77]. Laser photocoagulation, instead, is more incisive, but, on the other hand, can cause infectious ulcerative keratitis [78].

Pharmaceutical treatments consist of anti-VEGF drugs that can be administered intravitreally, by an injection that is less invasive than surgery. However, many studies reported collateral effects on retina vessels, i.e., intravitreal angiogenesis, retinal detachment and a-vascularised retina [66,79]. On the other hand, administration of BH4 partially corrects the hyperoxia-induced endothelial dysfunction through rescuing the physiological levels of eNOS activity [80].

Finally, animal model studies showed that a possible therapeutic approach is to stimulate progenitor cells to promote retina neovascularization. Endothelial progenitor cells are more numerous and more accessible compared to stem cells and, as mentioned before, they are able to activate angiogenesis and quiescent endothelial cells. Among the EPCs’ different subpopulations, the endothelial colony-forming cells (ECFCs or late EPCs) are the most promising candidates for this kind of treatment. These cells contribute to angiogenesis and vasculogenesis via incorporation in developing vessels. During ECFC reperfusion, macrophage infiltration, oxidative stress and tubular necrosis are reduced [81]. On the other hand, ECFC can also promote tumor initiation and metastatic formation [82].

A last, very interesting approach is the use of progenitor cell-derived soluble factors. Instead of supplying EPCs, these substances can activate cell mobilization toward the tissue site where the activation of reparative vascularization and tissue perfusion is needed [83].

By improving EPC homing, LAV-BPIFB4 therapy may prevent micro-vasculopathies. Furthermore, micro-vasculopathies are important predictors of subsequent macro-vasculopathies, and an early intervention may significantly improve cardiovascular health.

## 9. Conclusions

Aging is a major risk factor for the onset and development of CVDs, the leading cause of death in industrialized countries. Indeed, aging results in compromised cellular and molecular events governing the endothelial and the immune compartment and their functional interplay in CVDs’ onset.

Healthy LLIs are genetically predisposed to delayed aging and age-related diseases and hence they can be considered a good model to identify the keys that can guarantee a long healthy life. Different alleles have been associated with longevity, demonstrating that a favorable genetic background is able to protect from aging. Among these, LAV-BPIFB4 has been shown to counteract the decline of endothelial function through the rescue of NO levels, the improvement of EPC homing and the enhancement of calcium mobilization. These beneficial effects constitute innovative perspectives for both micro- and macro-vasculopathy treatments.
